# Image processing for identification and quantification of filamentous bacteria in in situ acquired images

**DOI:** 10.1186/s12938-016-0197-7

**Published:** 2016-06-11

**Authors:** Philipe A. Dias, Thiemo Dunkel, Diego A. S. Fajado, Erika de León Gallegos, Martin Denecke, Philipp Wiedemann, Fabio K. Schneider, Hajo Suhr

**Affiliations:** Graduate Program in Electrical and Computer Engineering, Federal University of Technology Paraná, Av. Sete de Setembro 3165, Curitiba, 80230-901 Brazil; Department of Information Technology, Mannheim University of Applied Sciences, Paul-Wittsack-Str. 10, 68163 Mannheim, Germany; Institute for Urban Water and Waste Management, University of Duisburg-Essen, Universitätsstr. 15, 45141 Essen, Germany; Department of Biotechnology, Mannheim University of Applied Sciences, Paul-Wittsack-Str. 10, 68163 Mannheim, Germany

**Keywords:** Filamentous bacteria recognition, Digital image processing, Filamentous bulking and foaming, In situ microscopy, Filamentous microorganism, Wastewater treatment

## Abstract

**Background:**

In the activated sludge process, problems of filamentous bulking and foaming can occur due to overgrowth of certain filamentous bacteria. Nowadays, these microorganisms are typically monitored by means of light microscopy, commonly combined with staining techniques. As drawbacks, these methods are susceptible to human errors, subjectivity and limited by the use of discontinuous microscopy. The in situ microscope appears as a suitable tool for continuous monitoring of filamentous bacteria, providing real-time examination, automated analysis and eliminating sampling, preparation and transport of samples. In this context, a proper image processing algorithm is proposed for automated recognition and measurement of filamentous objects.

**Methods:**

This work introduces a method for real-time evaluation of images without any staining, phase-contrast or dilution techniques, differently from studies present in the literature. Moreover, we introduce an algorithm which estimates the total extended filament length based on geodesic distance calculation. For a period of twelve months, samples from an industrial activated sludge plant were weekly collected and imaged without any prior conditioning, replicating real environment conditions.

**Results:**

Trends of filament growth rate—the most important parameter for decision making—are correctly identified. For reference images whose filaments were marked by specialists, the algorithm correctly recognized 72 % of the filaments pixels, with a false positive rate of at most 14 %. An average execution time of 0.7 s per image was achieved.

**Conclusions:**

Experiments have shown that the designed algorithm provided a suitable quantification of filaments when compared with human perception and standard methods. The algorithm’s average execution time proved its suitability for being optimally mapped into a computational architecture to provide real-time monitoring.

## Background

Among the different problems that can occur throughout activated sludge processes in wastewater treatment plants (WWTP) and thus hinder proper sludge settling, two of them are commonly caused by filamentous bacteria: bulking and foam formation [1]. These microorganisms are part of the activated sludge biocenosis and considered to create the backbone for sludge flocs, but problems can arise when they outcompete the floc-forming bacteria under specific conditions [2]. Typically, concentrations of filamentous bacteria have been monitored by means of human-performed, off-line and time demanding methods based on optical microscopy. Since these techniques are intrinsically subjective and susceptible to human errors, automated image analysis tools have been used for monitoring activated sludge processes [3].

Costa et al. [3] provide an overview of the techniques reported in the literature for quantitative image analysis of wastewater environments. Typically, filaments and aggregates are identified using:

- Phase-contrast microscopy—images with bright flocs and dark filaments, allowing a classification via direct brightness thresholding [4, 5];
- Fluorescence microscopy—combined with staining techniques, provides images where each type of biological structure (e.g. flocs and filaments) is represented with a specific color [6];
- Bright-field microscopy—filaments and flocs appear as dark objects against a brighter background, requiring morphological analysis for their distinction [7, 8].

Some studies combine different imaging methods for characterization of each structure. In general, bright field microscopy is used for floc characterization, while phase contrast is applied for filaments analysis [5, 8, 9].

As a disadvantage, these analysis methods still make use of discontinuous microscopy, a practice that requires sampling, transport and sample preparation before evaluation. Besides being labor-intensive, information may be altered during this process. There is also no consensus on the minimum sample size and number of images required for proper evaluation of activated sludge [10]. The in situ microscopy appears as a promising technique for an accurate, rapid and sampling-free monitoring of the abundance of filamentous bacteria under real environment conditions [11]. It can be directly installed in bioreactors or pipelines, eliminating sample collection and preparation of slides. Moreover, it allows an analysis with low statistical error, since large amounts of independent images (i.e. each image is equivalent to a new slide) can be acquired for each sample [12].

Current in situ microscopes are based on bright field microscopy. As a consequence, image processing techniques different from the ones used with phase-contrast illumination and fluorescence microscopy are required for the identification of filamentous bacteria. First, segmentation between flocs and filaments through direct brightness thresholding (e.g. Jenne et al. [5] and Amaral et al. [9]) is not viable for in situ acquired images.

Algorithms proposed for bright-field microscopy, as the ones described by Motta et al. [7], Mesquita et al. [8] and Lee et al. [13], are also based on some characteristics that are not true in the case of in situ acquired images. Most of them are designed for discontinuous microscopy, where proper dilution can be performed in cases of high concentration and background images can be acquired and subtracted for image enhancement. This is also the case of the in situ system proposed by Koivuranta et al. [14], where samples are diluted before evaluation. Moreover, these works have typically employed median filtering for differentiation between flocs and filaments, calculated using large window size (e.g., 25 $\times$ 25 [13]). In contrast, we describe an approach based on the Euclidean distance transform for a more refined thickness determination.

This paper introduces an image processing method for identification of filamentous bacteria from in situ acquired images. Unlike the reviewed studies, this method is suitable for real-time evaluation of images without any staining, phase-contrast or dilution technique. Moreover, as highlighted by Khan et al. [10], most algorithms for image analysis of activated sludge are described shortly and superfluously. They are rarely evaluated in terms of sensibility, accuracy and commonly present pre-defined parameter values that are not justified. In the present manuscript, the proposed method is explained in details, including an adaptation of the concept of ROC curves for evaluation and optimization of the developed algorithm. Finally, the algorithm here introduced provides an estimation of total extended filament length using geodesic distance transform for both pruning and extent measurement.

## Methods

### Image acquisition

**Fig. 1:**
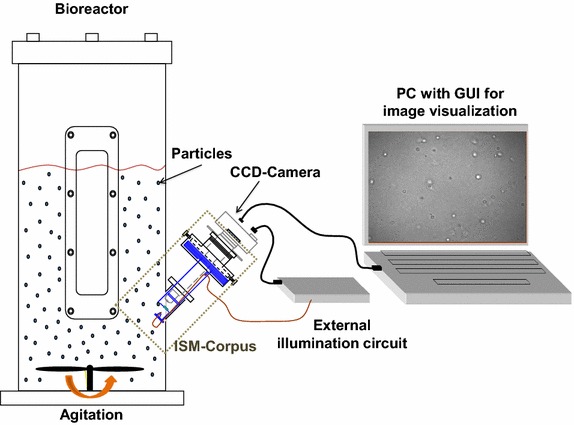
Typical configuration for experiments with the in situ microscope (ISM) developed at the Mannheim University of Applied Sciences

Images of the activated sludge environment were acquired with an in situ microscope (ISM) developed at the Mannheim University of Applied Sciences, sketched in Fig. 1. It is a pulsed transmitted light microscope, whose illumination is provided by a luminescence diode (DieMOUNT, Wernigerode, Germany) activated by an external circuit and guided inside the suspension via optical fiber [15]. Control pulses 0.5–10 μs wide ensure that, despite the speed of the microorganisms inside a bioreactor (0.1–1 m/s), they are still imaged without blurring [16].

The fiber-ending (cross section of 1 mm^2^) is positioned $\approx$0.3 mm above a quartz-glass window separating objective and suspension. The objective ($\times$40, NA = 0.75) is attached to the top of an internal tubus optically coupled to the window by means of water immersion. A CCD-Camera (Basler A102f) is attached to other extremity. Up to 10 monochromatic images (8-bit, no compression) per second can be acquired, with a resolution of 1392 $\times$ 1040 pixels (pixel size 6.45 $\times$ 6.45 μm^2^) [15]. A software is responsable for the control of all the system components, triggering both camera and pulse generator according to frequency, gain and brightness defined via an user interface.

The $\times$40 nominal magnification is ensured by the ISMs tube length of 160 mm, so that for the given resolution and pixel size an 0.17 $\times$ 0.22 mm^2^ object field is captured. A virtual sample volume is purely optically defined by depth of focus, without need of any mechanically moving part [12]. The lateral width of the virtual sample volume is directly given by the boundary of the image field (i.e., 0.17 $\times$ 0.22 mm^2^). As described by Suhr et al. [12], its third dimension (i.e. its thickness) is defined by depth of focus, estimated by other applications to be approximately 10 μm [16]. For this work, it is not necessary to calibrate the sample volume. Instead, it is required that the same virtual sample volume is evaluated for all images. This is ensured by using always the same image processing parameters.

### Experimental set-up

Samples were weekly collected from an industrial activated sludge plant in Leverkusen, Germany (Currenta GmbH & Co OHG), from the second biological treatment step (cascade biology) and analyzed for a total period of twelve months. Results and further details of these experiments are explained in [11]. For these initial experiments, the in situ microscope (ISM) was used off-line. Aiming at a set-up that properly replicates real environment conditions, samples were directly introduced into a beaker, without any prior conditioning as dilution or staining. Inside this glass container, the ISM front-end sensor head was submerged and, for each sample, 500 images were acquired at a frequency of 3 images/second. Given the small virtual sample volume defined and since a magnet stir at 300 rpm kept the suspension agitated, all images are independent from each other, containing entirely new optical samples. As exemplified by Belini et al. [16], at a flow speed of 0.1 m/s the samples are exchanged about 300 times per second.

### Image processing methodology

**Fig. 2:**
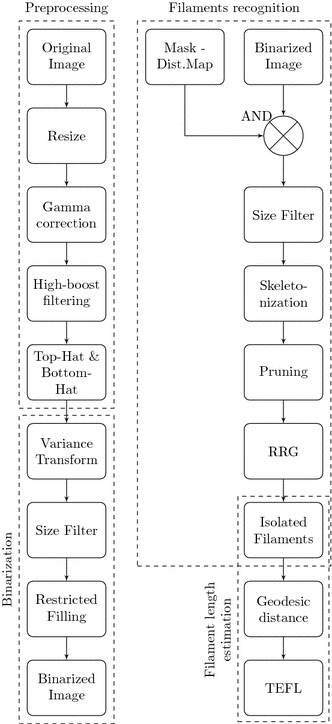
Flowchart of all operations performed in an ISM-image for filament detection and estimation of its length

The image processing algorithm here described was implemented in MATLAB 8.1 (The MathWorks Inc., Natick, MA, USA). ISM-images taken from wastewater contain not only filaments, but also other agglomerated objects. Since this study focuses on the analysis of filamentous bacteria, an algorithm able to recognize such thin, elongated patterns is necessary. The sequence of operations applied to each image is presented in Fig. 2.

#### Preprocessing

In order to save computational time, the image is first resized by a factor of two in both directions (1392 $\times$ 1040 to 696 $\times$ 520 pixels), using bicubic interpolation. Since the ISM provides resolution of 0.5 μm, even after image reduction the minimal filaments diameter can still be imaged with at least 2.5 pixels, so that there is no major loss of details. Actually, the bicubic interpolation also has an effect equivalent to a low-pass filter, contributing to eliminating some small noises in the image. An average computing time of approximately 0.7 s (around 3.7 times faster than non-resized images) in an Intel Core i7, 3.07GHz, 6GB RAM is achieved. This allows the analysis of thousands of filaments in a short measurement time.

Further, it was verified that an algorithm for Gamma correction according to the Eq. (1) provides a better encoding of grayscale values for identification of smaller transitions [17].

1 $$\begin{aligned} g = f^\gamma = f^{0.6} \end{aligned}$$

In Eq. (1), *f* is the input image and *g* is the output of this step. The ISM images contain filaments and flocs from clustered filaments and other material. Objects are darker in the image if they are in focus or slightly off focus in the direction of the objective. If they are off focus in the other direction, they appear brighter than the background. Moreover, filaments are imaged as thin structures whose identification resembles problems of edge detection, so that contrast and high-frequency components in both directions (i.e. darker than background and lighter than background) must be considered for segmentation.

In the present algorithm, these transitions are enhanced in two stages. First, a high boost sharpening filter is applied, where high-frequency components are emphasized without removing low-frequency ones. This is performed by adding the original image to its high-pass filtered version. After that, a combination of top and bottom-hat operations (structuring element SE: diamond, radius 3) is applied, expressed as:

2 $$\begin{aligned} \widehat{g} = \left( g + g \hat{\circ } SE \right) - g \hat{\bullet } SE \end{aligned}$$

In Eq. (2), *g* is the input and $\widehat{g}$ the output of this step. The top-hat operation ($\hat{\circ }$) is defined as the subtraction of the original image by its morphologically opened version, so that sharp bright peaks are enhanced [18]. In an analogous manner, the bottom-hat operation ($\hat{\bullet }$) consists of the subtraction of the original image from its morphological closing, in such a way that dark valleys are sharpened.

#### Binarization

After image enhancement, the segmentation between objects and background is performed using the concept of variance transform. Defined as the square root of the standard deviation, this statistical parameter is calculated in 3 $\times$ 3 windows for the original gray values. A variance transformed image is obtained, where regions of transition are highlighted [17]. This way, the virtual sample volume is ultimately defined by thresholding the variance transformed image with a fixed, optimal threshold value. Objects outside this range (i.e. out-of-focus and poorly focused objects) are imaged with so much blur that they do not pass this threshold and are therefore discarded. The resulting binary image contains the objects in focus present in the original image and some small debris, which can be removed by means of size filter.

According to reference data in [11], the most common species of filamentous bacteria present in the activated sludge under investigation (e.g. *M. parvicella*, *Type 1863*, *Type 0092*, *Type 0914*, *G. amarae*) have diameters of 0.8–1.2 μm and lengths of 10–200 μm [19]. Considering the ISM’s 40× magnification and its pixel length of 6.45 μm, the smallest diameter corresponds to 2.5 pixels in the final image:

$$\begin{aligned} \textit{diameter:}\, 0.8 \,\upmu \text{m} \, \xrightarrow []{\times 40} 32 \,\upmu \text{m}\, \xrightarrow []{\div 2\times 6.45 \,\upmu \text{m}} \,\approx\, 2.5\, \textit{pixels} \end{aligned}$$

For a rough estimation of area, filaments can be approximated as a rectangle whose area corresponds to the width multiplied by the length. Considering the smallest width and length expected, an analogous calculation indicates that true filaments must have areas larger than 77.5 pixels:

$$\begin{aligned}&\textit{length:}\, 10\,\upmu {\text{m}} \xrightarrow []{\times 40} 400\,\upmu {\text{m}} \xrightarrow []{\div 2 \,\times \,6.45\,\upmu {\text{m}}} \approx 31\, \textit{pixels} \\&\textit{area: diameter} \times { length} = 31 \times 2.5 \,\,\approx 77.5 \,\textit{pixels} \end{aligned}$$

**Fig. 3:**
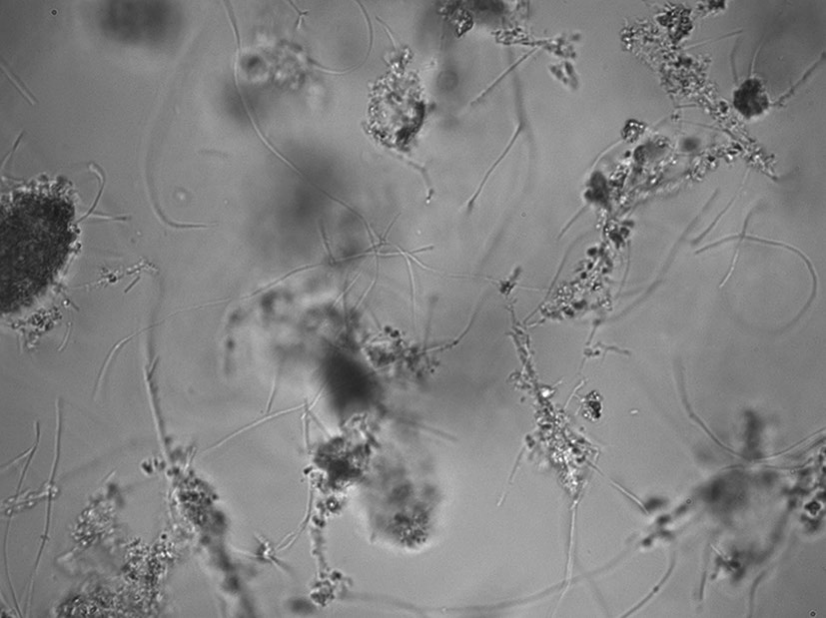
Example of ISM image of filamentous bacteria in activated sludge (industrial WWTP-Leverkusen)

**Fig. 4:**
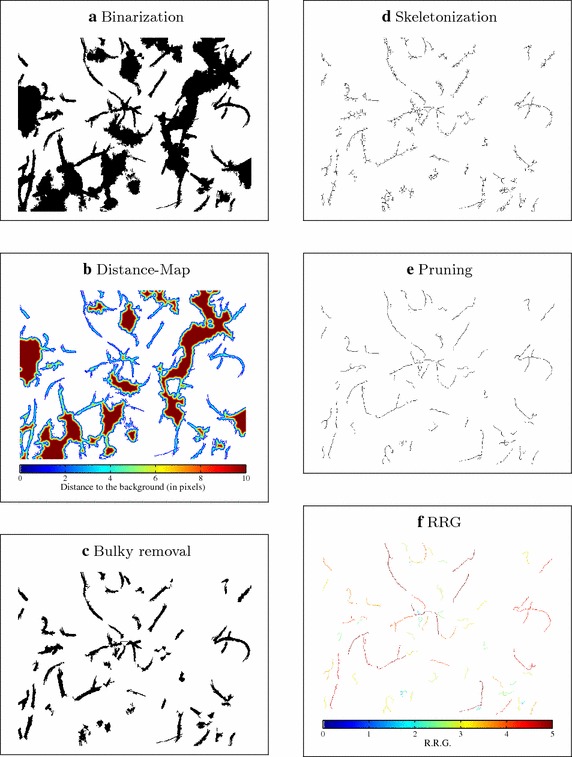
Images illustrating the sequence of operations for filament identification. **a** Image obtained after binarization. **b** Colormap of euclidean distance-mapping, used as measure of thickness. **c** Agglomerates identified in the previous step are removed from the original binarized image. **d** For better morphological characterization, the remaining objects are skeletonized. **e** A pruning operation using geodesic-distance transform is performed for isolation of spines. **f** Colormap of RRG, which provides a measurement of elongation for debris elimination

Thereafter, all objects with area smaller than 77 pixels are removed. Moreover, after binarization both filaments and agglomerates contain some small holes not present in the original image, so that these structures need to be filled for better approximation of their original forms. However, filaments can be curved and as a consequence form enclosed regions, so that a process of restricted filling is applied: only holes with diameter smaller than the ones expected from true filaments (i.e., 2.5 pixels) are filled. Finally, for an original image illustrated in Fig. 3, a binarized image as the one shown in Fig. 4a is achieved.

#### Filaments identification

*Distance mapping* In the absence of specific staining or phase-contrast differences, thickness features are used for distinguishing between filaments and other objects. For thickness determination, the distance transform is applied. A number is assigned to each black pixel of the binary image, corresponding to the euclidean distance between that pixel and the nearest white pixel composing the background, as illustrated in Fig. 4b.

A proper distance filtering, using a fixed, optimal threshold value, provides a binary image containing only agglomerates, somewhat eroded since their borders are also removed together with filaments and other small objects. After a suitable dilation to reconstruct their original form, a binary mask containing only flocs is obtained. By means of simple binary *AND* operation, the floc regions are removed from the original binary image (Fig. 4a), resulting in an image with only thin components as illustrated in Fig. 4c.

*Skeletonization* At this point, only filaments and some debris, typically parts from flocs, remain in the image. The two groups can be differentiated through measurement of elongation, since the filamentous bacteria present in wastewater are mostly elongated objects which do not exhibit many directional changes. Yet, a proper evaluation of such characteristic shall focus on the object’s spine, i.e. the medial axis along its skeletonized version. For this reason, first a skeletonization is performed by successively thinning the objects, removing boundary pixels without allowing connected parts to break apart. Figure 4d shows the output of this step.

**Fig. 5:**
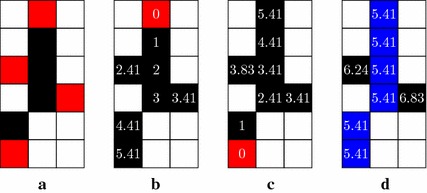
Spine recognition using geodesic distance.** a** *Red pixels* illustrate the four endpoints verified for computation of spines;** b**,** c** geodesic distances to each skeleton’s pixel, starting from endpoints marked in* red*;** d** spine (in* blue*) identified by local regional minimum

The skeletonization process yields objects spines together with their branches, which are unwanted for proper length and elongation measurements. For their isolation, we define spines as the shortest path between the two most distant endpoints of a skeleton, as suggested in [18]. Initially, each object is individually recovered in the smallest rectangular frames containing them. After that, each frame is processed to find out which pair of their endpoints has the largest distance from each other. In order to reduce the computational cost, the four endpoints nearest to the four boarders of the frame are preselected as possible candidates, as shown in Fig. 5a. If two or more endpoints touch a border, the one with smaller pixel index is chosen, i.e. the one closest to the upper-left corner.

The procedure for elimination of extremities (pruning) is based on the concept of geodesic distance transform [18]. This particular distance transform calculates for each skeleton pixel the path-length between that given pixel and all the other skeleton pixels, traversing only the skeleton as exemplified in Fig. 5b. The distance between each connected black pixel is measured with the concept of *quasi-euclidean distance*, expressed as follows for the distance between an origin ($x_1,y_1$) and another point ($x_2,y_2$):

3 $$\begin{aligned}&\left| x_1 - x_2\right| + \left( \sqrt{2}-1\right) \left| y_1 - y_2\right| \text {, } \quad \left| x_1 - x_2\right| > \left| y_1 - y_2\right| \nonumber \\&\left( \sqrt{2}-1\right) \left| x_1 - x_2\right| +\left| y_1 - y_2\right| \text {, otherwise} \end{aligned}$$

Also known as the strongest geodesic ends (SGE), the two most distant endpoints are recognized by finding the maximal geodesic distance (i.e., the longest path) along the skeleton when starting from each of the four candidate points. Once the SGE are identified, the actual spine can be discriminated from its spurious branches by determining the shortest path connecting the SGE. The technique to identify the spine pixels is related to an algorithm attributed to C. F. Gauss [20] for calculating the sum of an arithmetic progression 1 + 2 + 3 +···+ n. The sums of two numbers are assigned to every pixel. The first summand is the length of the path to one of the endpoints (known as geodesic distance function), while the second summand is the path-length to the other endpoint. Figure 5b illustrates the distance values obtained using one endpoint as origin, while Fig. 5c illustrates it for the other endpoint. As result, all pixels belonging to the spine will carry the same sum-value which is precisely the geodesic path length between the two endpoints. Pixels outside the spine present larger values, so that the spine can be easily determined by regional minima identification, as shown in Fig. 5d.

For cases of filaments crossing each other other (e.g., 'X' format), all branches longer than 31 pixels (minimum expected filament length) are reprocessed using the same technique, providing proper pruning. Such situation can be observed for example, in objects in the right side of Fig. 4d, e.

*Reduced radius of gyration (RRG)* At this stage, some objects originated from edges of larger agglomerates still need to be filtered out. A probabilistic approach based on the fact that true filaments are frequently more elongated and have less vertices than thin objects originating from agglomerate edges is used, applying the reduced radius of gyration (RRG) to identify the true filaments [21]. The RRG is defined as

4 $$\begin{array}{*{20}{c}} {RRG = \frac{{\sqrt {{M_{2x}} + {M_{2y}}} }}\quad {{\frac{{{D_{eq}}}}{2}}}}&{{D_{eq}} = 2\sqrt {\frac{A}{\pi }} }\\ {{M_{2x}} = \frac{{\sum\nolimits_{i = 1}^N {{{\left( {{x_i} - {x_g}} \right)}^2}} }}{N}}&\quad {{M_{2y}} = \frac{{\sum\nolimits_{i = 1}^N {{{\left( {{y_i} - {y_g}} \right)}^2}} }}{N}}\\ {{x_g} = \frac{{\sum\nolimits_{i = 1}^N {{x_i}} }}{N}}&\quad{{y_g} = \frac{{\sum\nolimits_{i = 1}^N {{y_i}} }}{N}} \end{array}$$

In Eq. (4), the position of a pixel *i* of an object is $(x_i,y_i)$, $D_{eq}$ refers to the diameter of an circle with area equivalent to the object’s area A, N is the amount of object’s pixels and $(x_g,y_g)$ are the coordinates of the object’s center of gravity (centroid). The momentum in each dimension is calculated ($M_{2x}, \,M_{2y}$) to compute the RRG. For a perfect disc, the RRG is equal to $\sqrt{2}/2$.

**Fig. 6:**
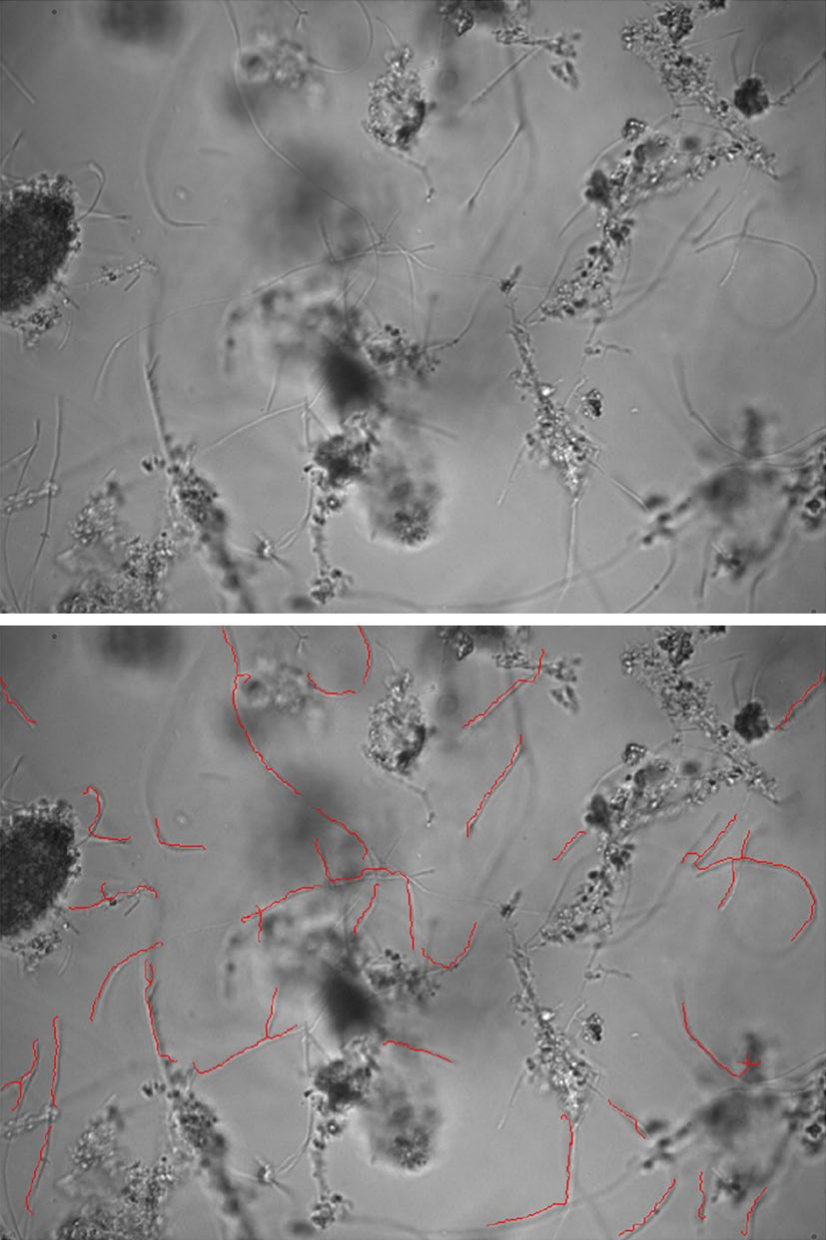
Comparison between original ISM image (*upper image*) and its version with detected filaments in* red*

Figure 4f provides a colormap illustrating the RRG values calculated for the detected pruned skeletons. Filaments are more elongated and therefore have larger RRG, so that via suitable thresholding using a fixed, optimal threshold value they can be isolated as shown in Fig. 6.

#### Estimation of filaments lengths

Once the filaments are identified, their total extended length can be estimated. This information is obtained from the path lengths between endpoints already measured during pruning steps. Thereafter, their real length dimensions is estimated by applying a scale to these distance values. Since each pixel represents an area of 6.45 $\times$ 6.45 μm^2^ and the image was resized by a factor of 2, the distance is first multiplied by 12.9. The ISM’s tube length of 160 mm ensures a nominal magnification equals to the objective specification, being thereafter necessary to divide the length by 40. The scale factor is summarized as

5 $$\begin{aligned} length = distance\times \frac{2\times 6.45}{40} [\upmu \text{m}] \end{aligned}$$

### Reference images

For evaluating the image processing algorithm, real ISM images were selected from sets acquired for different activated sludge samples. The chosen reference images cover different scenarios, emphasizing difficult ones, such as: mix of filaments well and poorly focused, some connected to grainy structures; filaments crossing each other and grainy structures; short and long filaments; high concentration of flocs.

For the establishment of the ground truth for filament detection, each reference image had its filaments marked by five researchers in the area of signal/image processing, who have a proper expertise to define which filaments are properly focused and should be detected by the algorithm. Filaments marked by three or more specialists were considered true filaments.

### Receiver operating characteristics (ROC) curves

The evaluation of detection rate was performed using the receiver operating characteristics (ROC) curves [22]. In the present study, pixels marked as filaments in the reference image are defined as positives (*P*), while all its other pixels are considered negatives (*N*). True positives (*TP*) stands for to pixels marked as filaments in the reference image that were correctly classified by the algorithm as filaments. On the other hand, false negatives (*FN*) pixels are the ones marked as filaments in the reference image, but not detected by the algorithm. Pixels neither identified by the algorithm as filaments nor marked in the reference image as such are defined as true negatives (*TN*). Finally, false positives (*FP*) denotes the pixels identified by the algorithm as filaments, but not marked in the reference image as such. The true positive rate (*TPR*) is computed using Eq. (6).

6 $$\begin{aligned} TPR = \frac{TP}{P} \end{aligned}$$

The FP rate (*FPR*) is usually computed as the rate between *FP* and *N*. However, for the present application it would correspond to all other objects in the image (e.g. flocs and debris) and the whole background region. Thus, calculating the FPR as a ratio of the total number of negatives would result in a very low rate, providing misleading conclusions about the algorithm specificity. To proper evaluate the error introduced by the recognition of false filaments, we opted for calculating the FPR as a ratio of the total number of positives, expressed as:

7 $$\begin{aligned} FPR = \frac{FP}{P} \end{aligned}$$

**Fig. 7:**
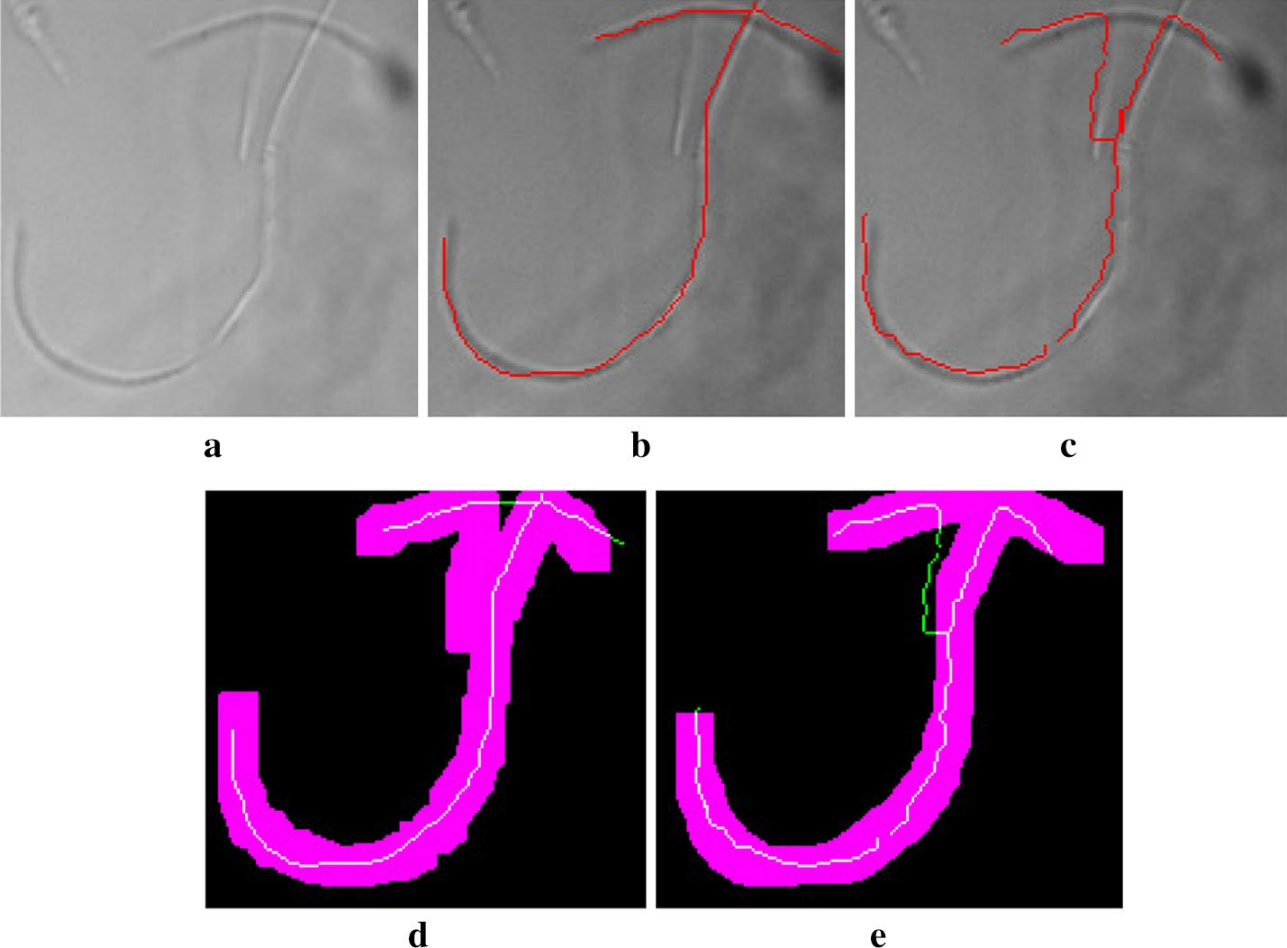
Steps for identification of pixels corresponding to TP, FN, TN and FP.** a** Original object;** b** filaments marked by experts;** c** filaments recognized by the proposed algorithm;** d** identification of TP (*white*) and FN (*green*);** e** identification of FP (*green*)

Similarly, specificity is defined as the complement of the FPR (i.e., 1-FPR). Figure 7 provides an example illustrating how TP, FN, TN and FP pixels are identified, for an object present in Fig. 7a. The pixels marked as reference are shown in Fig. 7b, while the ones detected by the algorithm are presented in Fig. 7c. The recognition of true positives and false negatives pixels is illustrated in Fig. 7d, where TP are in white and FN in green. It is performed through comparison between the reference markings and the dilated version of the algorithm detection, represented in purple. This dilation is necessary since small displacements can exist between detected filaments and respective reference ones. The recognition of false positives pixels, marked in green in Fig. 7e, is performed through comparison between the algorithm detection and the dilated version of the reference markings.

## Results and discussion

The developed algorithm aims at a quantification of filamentous bacteria and a estimation of their respective total extended filament length. To verify its applicability, comparisons to two different references techniques were performed.

### Hit-ratio of filament detection

Aiming at an isolated evaluation of the image processing itself, without influences of bad imaging (i.e. low quality images due to configuration mistakes) or errors intrinsic to the current reference methods, the hit-ratio of filament detection was computed using as reference twenty pre-marked images. Using the methodology presented for computation of TP, FP, TN, FN parameters, pixelwise comparisons between reference filament markings and the ones identified by the algorithm were performed.

**Fig. 8:**
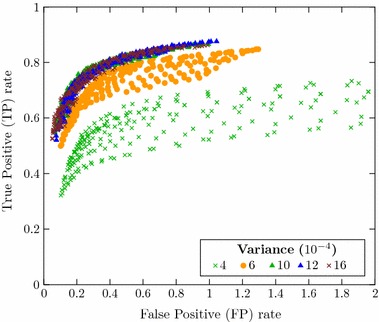
ROC curve with different combinations of variance, distance and RRG threshold values.* Markers/colors* are assigned according to variance threshold. Combinations with a common variance threshold form well-defined groups, so that the variance corresponds to the algorithm’s coarse adjustment

Since the performance of the algorithm for detection of filaments might be dependent on the threshold values applied for variance (binarization/definition of virtual sample volume), distance and RRG (finer adjustments), an optimization of threshold values was carried out. This was performed through analysis of ROC curves. Each point of the ROC curve shown in Fig. 8 represents a different combination of variance, distance and RRG thresholds. This chart illustrates how the variance threshold value acts as the coarse adjustment for the algorithm’s hit-ratio. Points corresponding to combinations with a common variance threshold value form well-defined groups.

**Fig. 9:**
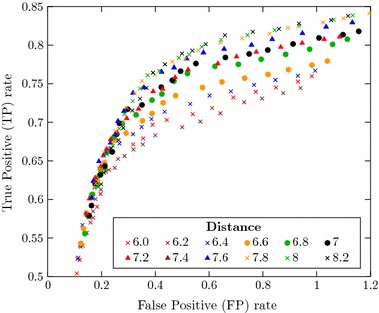
ROC curve for variance threshold $6\, \times \, 10^{-4}$. Combinations with common variance and distance threshold values form well-defined curves, being the distance threshold a finer adjustment

**Fig. 10:**
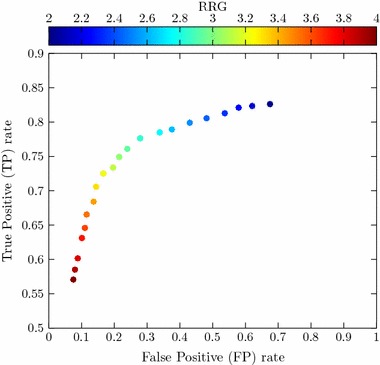
ROC curve for fixed variance and distance threshold values ($10 \, \times \, 10^{-4}$ and 6.4, respectively), with a colormap illustrating different RRG threshold values. The RRG acts as the algorithm’s final adjustment

Meanwhile, the values of distance and RRG thresholds correspond to finer adjustments inside each of these groups. As illustrated in Fig. 9 for one variance group ($6 \, \times \,10^{-4}$), combinations sharing common distance threshold values are distributed in form of well-defined curves inside each variance group. Finally, the Fig. 10 shows that the values of RRG threshold correspond to a latter fine adjustment which defines the system’s sensibility at the expense of a respective specificity.

**Fig. 11:**
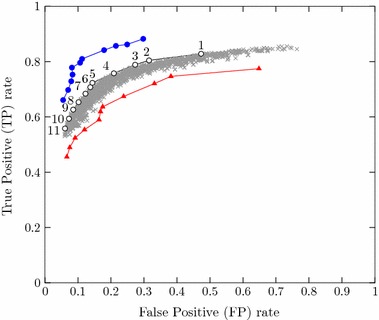
ROC curve of algorithm’s hit-ratio with the best combinations of variance, distance and RRG threshold values. Optimal points along the curve’s convex-hull are illustrated in* black*, with numbers assigned for matching with Table 1. In* blue*, results obtained for the ten images which yielded TPR higher than the median; in* red*, results obtained for the images that yielded TPR lower than the median

The ROC curve in Fig. 11 contains only points of optimal combinations. Points composing the curve’s convex-hull are highlighted, with their respective thresholds, TPR (equivalent to sensitivity), FPR, and specificity ratios shown in Table 1. In addition, the same chart also illustrates the hit-ratio obtained for two different groups: in blue, the results obtained for a group composed by the ten images for which the TPR values obtained were higher than the median; in red, values obtained for the group composed by images that yielded TPR lower than the median.

**Table 1:** Combinations of thresholds composing the convex hull of the ROC curve

Point	Var. ($10^{-4}$)	Dist.	RRG	TPR (%)	FPR (%)	Specificity
1	12	7	2.6	82.82	47.31	0.5269
2	9.5	8	3.1	80.42	31.54	0.6846
3	9.5	8	3.2	78.87	27.33	0.7267
4	10	7	3.2	75.77	20.88	0.7912
5	10.5	6.4	3.2	72.31	14.38	0.8562
6	10.5	6.3	3.2	70.74	13.74	0.8626
7	11.5	6.6	3.4	68.39	12.29	0.8771
8	10.5	6.3	3.5	65.33	10.19	0.8981
9	9.5	6.4	3.7	62.63	8.56	0.9144
10	9.5	6.4	3.9	59.34	7.28	0.9272
11	9.5	6.2	4	55.80	6.07	0.9393

Results revealed that, for these reference images, the automated analysis in average correctly detected up to 72 % of the pixels marked by specialists as filaments, with a false positive ratio equivalent to 14 % of this value (point 6 in Fig. 11). This corresponds to a specificity of about 86 %. Moreover, as illustrated by the blue and red curves, the true positive ratio oscillates between 64 and 81 % for this optimal combination of thresholds, with a false positive ratio between 11 and 17 %.

### Total extended filament length (TEFL)

One of the standard methods for quantification of filaments is the total extended filament length (TEFL), calculated according to Sezgin et al. [23]. The relationship of the TEFL with the SVI was first investigated by Sezgin et al. [23], Palm et al. [24] and Lee et al. [25]. These studies underline the influence exerted by filamentous bacteria on the sludge-settling behavior. For comparison with the reference TEFL method, 500 images from each wastewater sample were acquired and processed, so that an average ISM-online TEFL (or ISM-oTEFL) per image was computed by the image processing algorithm here proposed.

**Fig. 12:**
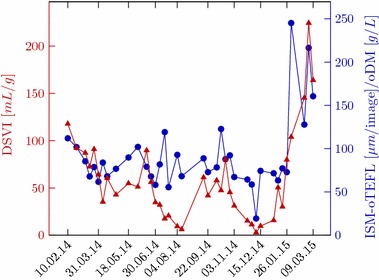
Comparison between conventional dSVI (*red*) and ISM-oTEFL/oDM (*blue*) Adapted from [11]

As illustrated in Fig. 12 and described by Dunkel et al. [11], a correlation between ISM-oTEFL/oDM and the diluted sludge volume index (dSVI) [26] is verified with a coefficient of r = 0.7. The diluted sludge volume index (dSVI) is a modification mentioned in Jenkins et al. [19], performed because the sludge samples were not able to settle below a volume of 200 mL during conventional SVI analysis. The organic dry matter (oDM) was measured according to DIN EN 12879. The suitability of the developed system for monitoring sludge settling properties was also supported by a Granger causality test, which yielded a p value of 0.0026 for the relationship of ISM-oTEFL and the dSVI. This situation supports the applicability of the proposed in situ quantification, since high quality images were obtained even for such situations of high MLSS-concentration (up to 8.3 g L^−1^ [11]), where the suspensions were totally black or brown and opaque if looked at them with the bare eye.

**Fig. 13:**
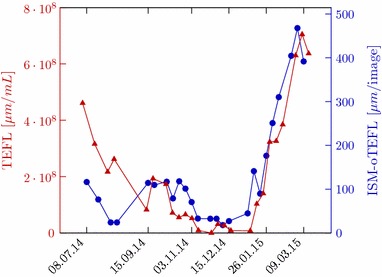
Comparison between conventional TEFL (*red*) and ISM-oTEFL (*blue*). Adapted from [11]

Moreover, as described by Dunkel et al. [11] and illustrated in Fig. 13, a linear relationship between the two methods is indicated by a Pearson correlation coefficient of r = 0.87. That means the proposed system is capable of detecting filamentous bacteria with good correlation to the reference TEFL method, specially in terms of trends over time, which is the critical information for decision taking against overgrowth of filamentous bacteria in activated sludge.

## Conclusion

The current typical methods for quantification of filamentous bacteria are based on discontinuous microscopy and visual analysis by human operators. As a consequence, these techniques are labor-intensive, susceptible to human errors and usually a limited number of images is analyzed. The application of in situ microscopy appears as a suitable tool for a more reliable, consistent monitoring of filamentous bacteria in wastewater treatment plants. Since no staining or phase-contrast techniques are employed, a new image processing algorithm was proposed for identification of filaments and estimation of their respective lengths.

Commonly, works describing image processing and analysis techniques for activated sludge samples are very brief and do not justify some pre-defined values. In this present work, an algorithm for identification of filamentous and estimation of their total extended length was described and evaluated using the concept of ROC curves. Moreover, parameters were justified and optimized using this metric.

Experiments have shown that the developed image processing algorithm can detect around 72 % of the amount of pixels marked by specialists as filaments in reference images, with a false positive rate of 14 %. Moreover, it is capable of detecting filamentous bacteria with good correlation to the reference TEFL method, specially in terms of trends over time, which is the critical information for plant operators actions against overgrowth of filamentous bacteria in activated sludge. With an average execution time of 0.7 s per image, the algorithm is therefore suitable for being optimally mapped into a computational architecture to provide real-time monitoring.

Future work includes evaluation under real conditions by attaching an in situ microscope into a pilot wastewater treatment plant, avoiding possible errors caused by sampling, storage and transporting. In addition, real-time analysis could be carried out, facilitating correlations to the other standard biological indicators. Furthermore, the ISM can be used in the future as an automatic controlling tool for specific counter measures against bulking and foaming.

For the ISM used in this work, only microbial aggregates smaller than 0.3 mm can pass through the gap between the fiber-ending and the quartz glass window separating objective and suspension. For experiments realized up to now, microscopic analyses have shown that the samples evaluated were composed of mainly stable and compact sludge flocs, with maximal size of 241.6 μm [27]. Apart from filamentous bacteria, the floc size, floc shape and stability of the sludge flocs influence sludge settleability. For this reason, further ISM studies will include these parameters in the image evaluation. If future experiments should prove that a bigger gap is necessary, both microscope and image processing algorithm can be adapted, since it would be a matter of brightness/gain and thresholds adjustments.

Finally, the proposed algorithm has also room for improvements, mainly for cases such as short crossing filaments, which are eliminated by the RRG filter. Besides, closely spaced filaments are sometimes binarized as one single massive object, further removed by the distance filter.

## Abbreviations

WWTP: wastewater treatment plants
ISM: in situ microscope
SGE: strongest geodesic ends
RRG: reduced radius of gyration
TEFL: total extended filament length
ROC: receiver operating characteristics
TP: true positive
FP: false positive
TN: true negative
FN: false negative
TPR: true positive rate
FPR: false positive rate
SVI: sludge volume index
MLSS: mixed liquor suspended solids
oDM: organic dry matter
